# Analysis of Alzheimer's disease severity across brain regions by topological analysis of gene co-expression networks

**DOI:** 10.1186/1752-0509-4-136

**Published:** 2010-10-06

**Authors:** Monika Ray, Weixiong Zhang

**Affiliations:** 1 Washington University School of Engineering, Dept of Computer Science and Engineering, Saint Louis, MO 63130, USA; 2 Washington University School of Medicine, Dept of Genetics, Saint Louis, MO 63110, USA

## Abstract

**Background:**

Alzheimer's disease (AD) is a progressive neurodegenerative disorder involving variations in the transcriptome of many genes. AD does not affect all brain regions simultaneously. Identifying the differences among the affected regions may shed more light onto the disease progression. We developed a novel method involving the differential topology of gene coexpression networks to understand the association among affected regions and disease severity.

**Methods:**

We analysed microarray data of four regions - entorhinal cortex (EC), hippocampus (HIP), posterior cingulate cortex (PCC) and middle temporal gyrus (MTG) from AD affected and normal subjects. A coexpression network was built for each region and the topological overlap between them was examined. Genes with zero topological overlap between two region-specific networks were used to characterise the differences between the two regions.

**Results and conclusion:**

Results indicate that MTG shows early AD pathology compared to the other regions. We postulate that if the MTG gets affected later in the disease, post-mortem analyses of individuals with end-stage AD will show signs of early AD in the MTG, while the EC, HIP and PCC will have severe pathology. Such knowledge is useful for data collection in clinical studies where sample selection is a limiting factor as well as highlighting the underlying biology of disease progression.

## Background

The neuropathological hallmarks of Alzheimer's disease (AD) are the accumulation of extracellular amyloid plaques and intraneuronal neurofibrillary tangles (NFT) in the brain. Certain brain regions have shown increased susceptibilities to the pathological and metabolic characteristics of AD [1-5]. However, AD does not affect all brain regions simultaneously. Comparing the gene expression of the affected regions to identify the differences in the biological pathways perturbed in AD can lead to greater insight into its pathogenesis and progression.

Organising genes into co-expression networks helps in comparing biological phenomena across brain regions, and obtaining a global overview of the disease, which can enable us to further understand the disease. As is well known in Quantum mechanics, while the behaviour of particles is not well understood or intuitive at the quantum level, they do behave more intuitively at the macro level. Hence, the application of systems biology methods to understand complex diseases is crucial. Gene coexpression networks can provide a view of the relationship among genes, based on their gene expression profile, in a particular condition or time or disease. Genes in a coexpression network are connected to one another based on the similarity of their expression profiles. The rationale behind this is that coexpressed genes may participate in the same pathway or form complexes [6,7] that perform a specific function.

Numerous studies have analysed the similarities in network structures and coexpression clusters [8-10]. Recently, a study by Luscombe et al. showed that important biological knowledge can be gleaned from the differences in the network topology of condition-specific regulatory networks [11]. However, to the best of our knowledge, there have not been any analyses of the topological differences of coexpression networks and their interpretation in complex diseases. AD progresses in stages and is described in terms of incipient (Braak stages III-IV), mild/moderate (Braak stages IV-V) and severe AD (Braak stages V-VI) [12]. However, AD does not affect all brain regions simultaneously [13], and regions that are affected later in the timeline of the disease progression will reveal evidence of early AD pathology. With the recent deposition of laser captured microdissected microarray data from discrete brain regions affected in AD [1], we analysed the differential network topology to identify associations among the four different brain regions and the severity of AD.

**Figure 1:**
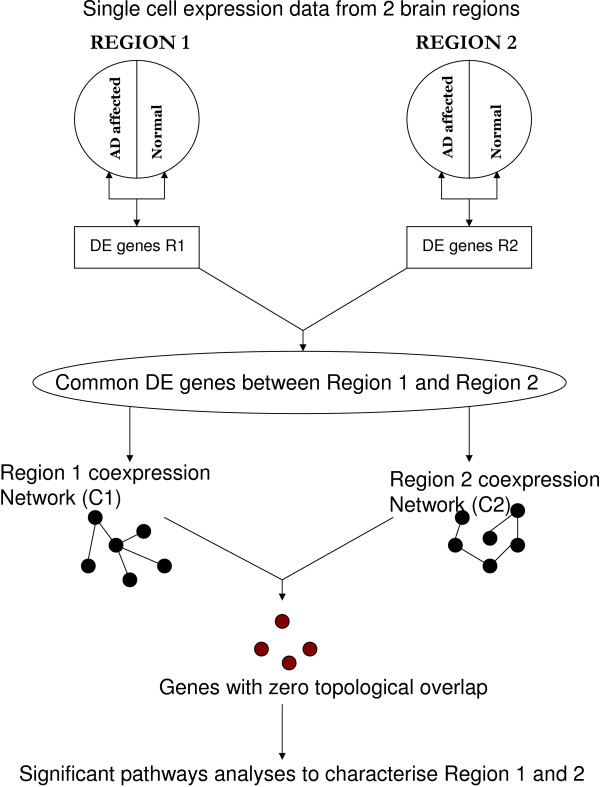
**Schematic of analyses carried out in this study**. This illustrates the flowchart of analysis performed in this study. R1 and R2 refer to region 1 and region 2, respectively. Differentially expressed (DE) genes were identified by comparing AD affected to normal controls within each region. When pairs of regions were compared, the coexpression network was built using the common (intersection) DE genes between the regions. Genes with no topological overlap between the coexpression networks were taken for further analyses.

In this study, our objective was to identify genes with differential topology in gene coexpression networks corresponding to different brain regions and observe the difference in AD severity across regions. We first identified the differentially expressed (DE) genes between AD affected and normal controls in the entorhinal cortex (EC), hippocampus (HIP), posterior cingulate cortex (PCC) and middle temporal gyrus (MTG). Then coexpression networks for the regions were built using the common DE genes between regions. Next, we investigated the topological differences between coexpression networks, and identified the significant biological pathways of the sets of genes with no topological overlap across the region-specific networks. The significant pathways along with network gene connectivity were used to determine the association between disease stage and brain region, and the relationship between gene activity and disease severity. Figure 1 shows the sequence of analyses undertaken in this study. Results suggest that the MTG is not as severely affected as the other three regions in this dataset. We provide further confidence in our analyses and results by comparing results from the MTG with those from the posterior visual cortex and superior frontal gyrus. Through illustration with AD relevant genes, we show that the change in connectivity of a gene can shed light on its behaviour in the disease stage. Furthermore, since we used laser captured microdissected expression data, we could compare different brain regions without any issues regarding regional variability due to cell type distribution. Such an analysis has implications for AD data collection, early AD detection and the identification of markers of early pathology.

## Methods

### Data

For our analyses we used recent microarray (Affymetrix Human Genome U133 Plus 2.0) expression data obtained via laser captured microdissection and contain data from different brain regions that are either histopathologically or metabolically relevant to Alzheimer's disease (AD) [1]. We used the data collected (mean postmortem interval of 2.5 hours) from the entorhinal cortex [EC; Brodmann area (BA) 28 and 34], hippocampus [HIP; CA1 region], middle temporal gyrus [MTG; BA 21 and 37], and posterior cingulate cortex [PCC; BA 23 and 31]. Alzheimer's affected subjects had a Braak stage ranging from III to VI [12] with a Consortium to Establish a Registry for Alzheimer's Disease (CERAD) neuritic plaque density of moderate or frequent [14]. For each sample, expression data was obtained from approximately 500 pyramidal neurons. The data consisted of 13 control subjects and 10 AD individuals for EC, 13 control subjects and 10 AD individuals for HIP, 12 control subjects and 16 AD individuals for MTG, and 13 control subjects and 9 AD individuals for PCC.

Probe sets were processed for differential expression (DE) by using the two-class significance analysis of microarrays (SAM) [15], based on GC Robust Multi-array Average (GC-RMA) summarised expression values [16]. SAM uses a modified *t*-statistics method to identify DE genes. Within each brain region, AD affected subjects and unaffected age-matched controls were compared to identify DE genes. After obtaining four sets of DE genes, one per brain region, we identified the common (intersection) DE genes between two regions of interest. The common genes were used to create two gene coexpression networks for the pair of regions from which the common genes were selected. These sets of common genes between regions will be referred to as the 'intersection genes' in the manuscript for clarity.

Significant biological pathways were identified using the well annotated GeneGo MetaCore™ database [17]. MetaCore™ is based on a proprietary manually curated database of human protein-protein, protein-DNA and protein compound interactions, metabolic and signalling pathways and the effects of bioactive molecules in gene expression [17].

### Construction of co-expression networks

We used the co-expression network (CoExp) method developed by Ruan and Zhang [18,19] to construct gene co-expression network by measuring the pairwise expression similarity between genes. Nodes in the network correspond to genes and edges represent expression similarities between genes. In this study, we used the Pearson correlation coefficient (PCC) for the similarity measure. For two genes to be considered as co-expressed, their expression profiles needed to satisfy at least one of the following conditions: (1) their correlation coefficient is higher than 0.3, and one gene is ranked as the top-3 most correlated gene of the other; (2) the correlation coefficient between them is higher than a user defined Pearson correlation coefficient threshold *t *(*t *= 0.7 or 0.8 in all the networks generated here) and one gene is within the top-50 most correlated gene of the other. According to the first condition, if 3 genes are correlated to gene A with correlation coefficients equal to 0.3, 0.32, and 0.4 then they get linked to gene A. On the other hand, if the 3 genes are correlated to gene A with correlation coefficients equal to 0.3, 0.28 and 0.29, then only 1 gene would get linked to gene A. However, this scenario rarely, if at all, occurs in gene expression data with a correlation coefficient threshold of 0.3 (see Additional file 1), hence the minimum connectivity in the networks in this paper is 3.

This approach was taken to create a sparse coexpression network. Minimally complex, sparse gene networks have been shown to be more robust to perturbations and may be a constraint in shaping the evolution of gene network complexity [20]. The CoExp method has been previously successfully applied to study Alzheimer's disease [21].

### Topological overlap between coexpression networks

A coexpression network was constructed for two brain regions under study separately using the differentially expressed genes that were common to both regions, i.e. the intersection genes (see Methods). Additionally, gene coexpression networks were also created using the DE genes obtained by comparing AD affected and normal controls in order to compare affected and controls within each region. The coexpression network obtained from CoExp is a binary adjacency matrix with 0 referring to no link between two genes and 1 corresponding to a link between the genes.

Let the two coexpression networks be referred to as *network1 *and *network2 *corresponding to brain region 1 and brain region 2, respectively. Since *network1 *and *network2 *were built using the intersection genes between regions 1 and 2, the nodes in both the networks are the same, although the connections among them are different. Let each node/gene in the network be denoted as *gene_i _*where *i *= 1, 2,...,*m *and *m *is the total number of nodes in the network.

Topological overlap between gene coexpression networks, *network1 *and *network2*, refers to the overlap of the genes connected to *gene_i _*in *network1 *and *network2*, i.e. overlap of the neighbourhoods of *gene_i_*. Let the genes connected to a *gene_i _*in *network1 *be referred to as *X *and those in *network2 *be referred to as *Y*. The connectivity or degree of *gene_i _*in *network1 *is *d*1_*i *_and that in *network2 *is *d*2_*i*_. The topological overlap (TO) for *gene_i _*between *network1 *and *network2 *is given by

(1) $$TO_{gen{\text{e}}_{i}}=\frac{X\cap Y}{max(d1_{i},d2_{i})}$$

The larger degree of *gene_i _*was considered instead of the smaller degree in order to reduce false negatives. Consider the following example. Let the neighbourhoods of *gene_i _*be *X *= 200 in *network1 *and *Y *= 10 in *network2 *with *X *∩ *Y *= 10, then with *max*(*d*1_*i*_, *d*2_*i*_) = 200, TO = 0.05. On the other hand, with *min*(*d*1_*i*_, *d*2_*i*_) = 10, TO = 1. Since our aim was to identify genes with a topological difference (i.e. low TO) between coexpression networks, this gene would have been discarded (false negative) if the smaller degree was used as it would result in a high TO value. The actual amount of similarity between these two neighbourhoods is only 5% (i.e. 10/200 unique genes). The TO values in this analyses were rounded upto 4 decimal places.

If the topology of coexpression networks are representative of the 'biological activity' of a gene under a certain condition or in a specific region, then genes with high topological overlap between two region-specific networks, may not differ greatly in their activity in the two brain regions. However, genes with low topological overlap probably have roles/activities that are region-specific or condition-specific. As a specific case for this analysis, genes with the maximum topological difference, i.e. TO = 0 (zero topological overlap since there are no overlapping genes between their neighbourhoods) between the two networks were selected for further analyses in this study. Genes with other values of topological overlap, if properly justified, can also be considered. Comparisons against 1000 random networks (random additions or deletion of links to the original network while keeping the degree of the genes equal to the original network) using *t*-statistics were made to assess the significance of the zero TO genes. The actual gene co-expression networks had a lower number of zero TO genes compared to the random networks. The significance values (*p *values) were calculated (with 999 degrees of freedom) using the following formula,

(2) $$\frac{\left|\mu_{1}-\mu_{2}\right|}{SD*\sqrt{\left(\frac{1}{n_{1}}+\frac{1}{n_{2}}\right)}}$$

where *μ*_1 _is the mean number of zero TO genes in the real network; *μ*_2 _is the mean number of zero TO genes in randomised networks; SD is the standard deviation of the number of zero TO genes in the 1000 random networks; *n*_1 _is the number of random nets (i.e. 1000) and *n*_2 _is the number of real networks (i.e. 1).

Some studies have used the topological overlap measure for identifying metabolites that are in the same functional class [22], or for module detection, i.e. clustering of genes [23]. Other network measures, not necessarily topological overlap, have been used for other objectives as in [8-11]. Although the topological overlap measure introduced in this article is similar to the ideas proposed in [22], it is still very different. The main difference lies in the determination of the topological overlap as it was motivated by very different biological objectives. While other topological measures were developed for the purposes of examining relationships among genes within the same network, our topological measure was developed to compare two different networks and use it to study the behaviour of a particular gene in separate gene coexpression networks that correspond to different brain regions. In this manner, the measure was used as a means of computing gene coexpression network differences and then associating these differences with AD severity. Such a use of a topological overlap measure has never been employed before.

## Results and Conclusions

### Genes with zero topological overlap between brain regions

Significance analysis of microarrays (SAM) [15] was used to identify differentially expressed (DE) genes between AD affected and unaffected controls within each of the four brain regions -en-torhinal cortex (EC), hippocampus (HIP), posterior cingulate cortex (PCC) and middle temporal gyrus (MTG) (Table 1). The analysis steps are shown in Figure 1. DE genes have been considered to be disease associated [24-27]. Other studies have used DE genes for class prediction [28-32].

**Table 1:** Number of differentially expressed (DE) genes and associated false discovery rate (FDR) in the four brain regions

Region	Number of DE genes (FDR)
Entorhinal cortex (EC)	5776 (0.5%)
Hippocampus (HIP)	5264 (0.5%)
Middle temporal gyrus (MTG)	3379 (0.5%)
Posterior cingulate cortex (PCC)	6536 (0.4%)

Six sets of intersection genes (see Methods) were obtained from six comparisons - (1) EC and HIP; (2) EC and PCC; (3) EC and MTG; (4) HIP and PCC; (5) HIP and MTG; (6) PCC and MTG (Table 2). Coexpression networks were built for each region using the intersection genes (see Additional file 2). For instance, the gene expression of the 2041 intersection genes between the EC and HIP were taken from the EC and used to build the coexpression network of the EC (ECnet), while the expression of the 2041 genes in the HIP were used to build the network corresponding to the hippocampus (HIPnet). Therefore, there were three different coexpression networks for each of the four regions resulting in twelve coexpression networks.

**Table 2:** Number of intersection genes, i.e. genes common to the set of DE genes of the regions being compared, and the number of genes with zero topological overlap

Regions compared	No. intersection genes	No. zero TO genes between regions
EC-HIP	2041	300
EC-MTG	1398	192
EC-PCC	2424	324
HIP-MTG	1248	271
HIP-PCC	3118	267
PCC-MTG	1582	180

The aim of this study was to identify the genes with differential topology between the region-specific coexpression networks. The biological processes represented by these genes would provide an idea of the difference, if any, in disease severity among the different regions. Therefore, after the coexpression networks were built for each region, the topological overlap (TO) between the region-specific networks was calculated for each pair of regions analysed. The numbers of genes with zero TO between the coexpression networks are shown in Table 2. These observed number of TO genes differ significantly from random expectation (*p *< 0.0001, see Methods). The entire list of these TO genes are provided as Additional file 3.

Although a few are mentioned here, there were many AD relevant genes in all the sets of zero TO genes (see Additional file 3). BR serine/threonine kinase 2 (*SAD1 *), calcitonin gene-related peptide-receptor component protein (*RCP9 *), calcitonin 1 (*CALCA*), cyclin-dependent kinase 2 (*CDK2 *), coronin actin binding protein 1B (*CORO1B*), interleukin 10 (*IL10 *), ninjurin 1 (*NINJ1 *), oxidative-stress responsive 1 (*OXSR1 *), phospholipase c eta2 (*PLCH2 *), spectrin beta non-erythrocytic 1 (*SPTBN1 *), plexin B2 (*PLXNB2 *), synapsin III (*SYN3 *), syntaxin 16 (*STX16 *), lim domain and actin binding 1 (*LIMA1 *), toll-like receptor 4 (*TLR4 *), yy2 transcription factor (*YY2 *), microtubule associated serine/threonine kinase 1 (*MAST1 *), were some of the AD associated genes in the list of 300 zero TO genes between the ECnet and HIPnet, that were associated with processes such as protein transport, cytoskeletal organisation, neurotransmitter release etc. [33-37]. *YY2 *is highly similar to the evolutionarily well-conserved zinc finger gene *YY1 *[38], which activates beta-site amyloid precursor protein-cleaving enzyme 1 (*BACE1 *) expression [39]. *BACE1*, which was present in the list of 271 genes between HIPnet and MTGnet, is necessary for the generation of beta-amyloid peptides, the principal constituents of senile plaques, in AD subjects [40,41]. Toll-like receptor 4 (*TLR4 *) signalling pathway has been implicated in the clearance of beta amyloid deposits in the brain of Alzheimer's disease subjects [42,43]. All three members of the caveolin gene family - caveolin-1, -2, and -3, were present in the set of zero TO genes, specifically in the list of 271 genes between MTGnet and HIPnet, 300 genes between the ECnet and HIPnet, and 324 genes between ECnet and PCCnet. This gene family has been implicated in AD, diabetes and cancer [44].

### Association between brain regions and disease severity

Table 3 lists the top few significant pathways, identified by GeneGo [17], in the six sets of zero TO genes. It was observed that the pathways represented by the zero TO genes could be broadly divided into two categories - inflammation/immune related pathways, and transport/cytoskeleton remodelling pathways.

**Table 3:** Significant biological pathways in the set of zero TO genes between regions.

Region comparison	Pathways	p value (FDR = 0.05)
EC-HIP	Bacterial infections in CF airways	1.27e-4
	Transcription NF-kB signalling pathways	1.46e-4
	Immune response MIF in innate immunity response	2.34e-4
	G-protein signalling RhoB regulation pathway	3.09e-4
	Immune response-bacterial infections	7.75e-4
	Toll-like receptor signalling pathway leading to proinflammatory response	9.02e-4
EC-MTG	TGF, WNT and cytoskeleton remodelling	4.51e-4
	Transport Rab-9 regulation pathway	1.48e-3
EC-PCC	Immune response-Function MEF2 in T lymphocytes	9.40e-4
	Plasmin signalling	3.51e-3
HIP-MTG	Transport Rab-3 regulation pathway	6.52e-3
	Intracellular cholesterol transport	6.56e-3
HIP-PCC	Neurophysiological process - Dopamine D2 receptor transactivation of PDGFR in CNS	9.64e-4
	TGF beta mediated regulation of cell proliferation	1.95e-3
PCC-MTG	Sorting endosome formation in CF	2.51e-4
	Role of inhibitor of apoptosis (IAP) proteins	1.23e-2

The EC has been shown to be the germinal site of AD followed by other brain regions [12,45]. Impairment of the transport ("Neuronal traffic jams") and cytoskeletal related processes in early AD pathogenesis has been shown by several studies [46-48]. Since the EC and HIP get affected earlier in the disease, subjects affected with severe AD, would have greater disruptions in the cytoskeletal, transport, energy metabolism, and lipid metabolism systems in these regions, resulting in secondary biological processes becoming highly active in the later stages of AD progression. Inflammation is a secondary process, i.e. a defensive reaction, that follows the perturbation of some other biological processes. Inflammation and cell death seem to be the final biological symptoms of AD. This is probably why the inflammation and immune response pathways were over-represented in the list of zero TO genes of regions that are afflicted with late AD pathology and overshadowed the biological pathways that triggered them.

On the other hand, transport and cytoskeleton pathways were highly significant, compared to other pathways, in the sets of 192, 271 and 180 genes obtained when MTG was being compared to the other regions. This implied that the inflammatory and immune responses were not yet dominant in the MTG. Furthermore, pathways that counteract cell death were probably active in MTG, since one of the significant pathways was the role of inhibitor of apoptosis proteins (Table 3), which suppresses apoptotic cell death. Since inflammation dominates other pathways in the later stages of AD [49,50], but such pathways were not highly significant in these sets of zero TO genes, as well as the fact that protective pathways were still highly significant, it can be postulated that the degenerative effects of AD in the MTG was less severe than that in the EC, HIP and PCC. From these results, we concluded that EC, PCC and HIP show pathogenesis of late stage AD while the MTG shows early AD pathology.

We hypothesised that the reason that transport and cytoskeletal related zero TO genes were showing up in MTG comparisons was probably because the source pool of genes, i.e. the set of intersection DE genes between the MTG and any other region contained mainly transport and cytoskeletal related genes. This meant that the MTG had more DE genes involved in transport and cytoskeleton compared to genes that were involved in inflammation and immune response. We reasoned that this situation could be because the MTG showed very early signs of AD.

The laser captured microarray dataset generated by Liang et al. included the microarray data of 6 regions - entorhinal cortex (EC), hippocampus (HIP), middle temporal gyrus (MTG), superior frontal gyrus (SFG), posterior visual cortex (PVC) and posterior cingulate cortex (PCC) [1]. Ac-cording to literature study in the report by Liang et al., the SFG shows metabolic changes relative to normal ageing and the PVC is spared from age-related and AD-related neurodegeneration [1].

It is conceivable that if a region is minimally affected by AD or AD-like (such as ageing) neurodegeneration, then inflammation or immune response related pathways will not be very statistically significant - as we have already reasoned that they are secondary processes in AD. When we analysed the DE genes of the SFG and PVC, we found this to be the case. While there were immune response pathways, there were many development and cytoskeletal related pathways. From these results we conclude that by using our method we can identify that the MTG does not seem to be as severely affected as the other 3 regions.

As a comparison, we identified the significant pathways of all the intersection genes (Number of genes shown in Table 2, column 2) in the six comparisons. Table 4 lists some of the top significant pathways of these intersection genes. Another comparison was analysing the differentially expressed genes between regions, i.e. DE genes that are AD relevant (i.e. comparing AD affected and controls within a region) as well as those that have differential expression across two regions. The top significant pathways, if any, are shown in Table 5. It could be seen from Tables 4 and 5, that there were a lot of similarities among the different regions and the differences between regions did not stand out as in Table 3. Such analyses and results are ineffectual in making any deductions about the disease stages in the regions.

**Table 4:** Significant biological pathways represented in the set of intersection genes between regions.

Region comparison	Pathways	p value (FDR = 0.05)
EC-HIP	NF-AT signaling in Cardiac Hypertrophy	6.711e-7
	Role of heterochromatin protein 1 (HP1) family in transcriptional silencing	3.309e-6
	NGF activation of NF-kB	9.559e-6
EC-MTG	Cytoskeleton remodelling Neurofilaments	3.564e-8
	Oxidative phosphorylation	1.314e-6
	Development-A2B receptor: action via G-protein alpha s	2.328e-5
EC-PCC	Role of heterochromatin protein 1 (HP1) family in transcriptional silencing	5.846e-7
	Cytoskeleton remodelling-TGF, WNT and cytoskeletal remodelling	7.951e-7
	Chemokines and adhesion	5.369e-6
HIP-MTG	Oxidative phosphorylation	1.615e-24
	Ubiquinone metabolism	2.971e-12
	Clathrin-coated vesicle cycle	3.490e-8
HIP-PCC	Oxidative phosphorylation	6.858e-13
	TGF, WNT and cytoskeletal remodelling	1.037e-7
	Role of 14-3-3 proteins in cell cycle regulation	2.839e-7
PCC-MTG	Oxidative phosphorylation	1.003e-32
	Ubiquinone metabolism	3.485e-16
	Clathrin-coated vesicle cycle	1.789e-8

Identification of differentially expressed (DE) genes was carried out by comparing AD affected and unaffected samples within a region. The intersection of the DE genes between two regions was extracted and significant pathways identified (See Table 2, column 2).

**Table 5:** Significant biological pathways in the set of differentially expressed genes between regions.

Region comparison (# DE genes)	Pathways	p value (FDR = 0.05)
EC-HIP (1349)	Ossification and bone remodelling	6.395e-3
	Cell cycle G2-M	8.544e-3
	Cardiac development FGF-ErbB signalling	9.847e-3
	Cell cycle-Mitosis	1.159e-2
EC-MTG (567)	Development	5.836e-5
	Transport-RAB3 regulation pathway	1.695e-4
EC-PCC (1685)	Role of 14-3-3 proteins in cell cycle regulation	1.188e-5
	Parkin disorder under Parkinson disease	1.474e-5
	TGF, WNT and cytoskeletal remodelling	2.738e-5
HIP-MTG (403)	Immune response-T cell receptor signalling	1.947e-6
	Immune response-Fc epsilon RI pathway	3.021e-6
	Dopamine D2 receptor transactivation of PDGFR in CNS	3.335e-6
HIP-PCC (259)	Immune response-Function of MEF2 in T lymphocytes	5.157e-8
	Immune response-CCR3 signalling in eosinophils	1.593e-6
	Immune response-Fc epsilon RI pathway	1.853e-6
PCC-MTG (210)	none at FDR = 0.05	-

Identification of differential expression was carried out across two brain regions by directly comparing the expression profiles of the two regions. The biological pathways of this set of differentially expressed genes between the regions was investigated and shown here.

We also examined the degree/connectivity of the zero TO genes in the coexpression networks (see Additional files 1 and 3). We found several AD associated genes that showed a marked difference in connectivity between the region-specific coexpression networks. Caspase-6 (*CASP6 *) was present in the list of 180 zero TO genes between the MTG-PCC, 271 genes between the HIP-MTG and 192 genes between the EC-MTG. *CASP6 *has been associated with early AD pathogenesis [51,52]. *CASP6 *was connected to 3 genes in the MTGnet and 11 in the HIPnet; 3 genes in the MTGnet and 24 in the PCCnet; and 21 genes in the ECnet and 6 genes in the MTGnet. Based on the large difference in connectivity between the region-specific networks, and the high connectivity in certain regions (EC, HIP and PCC) which we have previously suggested are severely affected, we hypothesised that *CASP6 *acts more aggressively in the later stages of the disease. *CASP6 *has been implicated in the early pathogenesis of Alzheimer's disease and continues to be an active participant in the later stages of the disease [52,53]. Furthermore, caspases are the principal executioners of apoptosis while the inhibitor of apoptosis proteins (IAP) are caspase inhibitors [54]. The IAP pathway was enriched in the set of 180 zero TO genes between the MTGnet and PCCnet. This further suggests that MTG shows early AD pathology. Beta secretase 2 (*BACE2 *) is implicated in AD [55,56] and was present in the set of 271 HIP-MTG zero TO genes. Increased levels of *BACE2 *is associated with early AD, and its expression is negatively correlated with disease progression [57,58]. It was connected to 24 genes in the MTGnet, compared to 3 genes in the HIPnet. *BACE2 *has been shown to be linked more to amyloid pathology than to neurofibrillary tangles pathology. This is supported by the evidence that it has a higher connectivity in the MTGnet, which is more susceptible to amyloid deposits than to neurofibrillary tangles [1]. Glutaminase 2 (*GLS2 *) was connected to 82 genes in the MTGnet and 17 genes in the PCCnet; 78 genes in the MTG and 3 genes in the HIP. Studies have shown large reductions in *GLS2 *activity in the brains of Alzheimer's subjects [59]. This is also evident from the lower number of connections of *GLS2 *in the PCC and HIP, a further indicator of PCC and HIP showing signs of late AD pathology. Brain-specific tubulin polymerisation promoting protein (*TPP/p25 *) had 60 neighbours in the MTGnet and 3 neighbours in the HIPnet. *TPPP/p25 *is an inhibitor of glycogen synthase kinase 3, thereby suppressing the phosphorylation of tau [60]. This competitive action fails during the evolution of tau pathology in the later AD stages. P21 activated kinase 3 (*PAK3 *) had 72 neighbours in the MTGnet and 3 in the HIPnet. Studies show that PAK3 levels were increased in early AD subjects, but declined in subjects with severe AD [61,62]. A few other examples of AD genes with a higher number of connections in MTGnet and lower connectivity in the other region-specific networks are insulin-like growth factor 2 receptor and cadherin 10. Studies have reported the decrease in expression of these genes with AD progression. Some genes with a lower connectivity in the MTGnet and higher connectivity in the other networks are caspase-3, semaphorin 3A, inducible nitric oxide synthase and nicastrin. The activity of these genes have been shown to be decreased in early AD pathogenesis. These results suggest that large differences in connectivity probably reflect the difference in the gene's activity in the different disease stages.

There were genes whose increased/decreased connectivity did not correlate with the in-creased/decreased activity in AD. However, in the majority of such cases, the temporal increase or decrease of a gene's expression in the course of the disease was not evaluated in the published reports. A gene's expression, although elevated from normal controls, can have a fluctuating response during disease progression. Such genes warrant further characterisation and our differential network method can aid in generating hypotheses related to this.

## Further remarks

The gene expression data analysed here was from the same organ and homogeneous cell population. Therefore, more similarities than differences are expected among brain regions with regard to AD affected pathways. However, identifying what differences, if any, are present among the AD affected regions may shed more light onto the disease progression. In this work, we presented a novel differential network topology method to examine four AD affected brain regions for differences in disease stages. This approach takes advantage of the logic that due to the progressive nature of the disease, different brain regions will be differentially affected and, therefore, would show different stages of AD progression/pathogenesis. We believe that such approaches that can investigate the differences across the affected brain regions from expression data collected from distinct brain regions are enabling tools for understanding AD progression and identifying signs of early pathology.

An alternate method of identifying significant pathways specific to a brain region, is to analyse the genes that are specifically expressed in the brain region, i.e. remove the genes that are common to two regions and analyse the genes unique to each region, instead of analysing the common genes. Our analyses using this approach didn't show marked differences between regions (data not shown). Furthermore, the same gene can have different functions depending on the physiological condition or brain region. Many genes have been known to play either destructive or protective roles depending on the condition, such as DNA repair genes allowing apoptosis or fighting against apoptosis. Furthermore, many genes also depict changes in their activity over the course of a disease. Our objective in this study was to analyse genes whose behaviour changed across regions, and determine if such changes in behaviour could characterise the difference in disease severity.

A natural step in this analyses could be the construction of gene co-expression networks using the *union of the DE genes *between two brain regions. In this case, some genes would be present in the network that were not considered differentially expressed in a certain region. Genes not considered significantly DE in any region could be due to 2 reasons - (a) noisy expression data and therefore not informative or (b) no change in expression in that region. We noticed that statistically significant DE genes with low gene expression tended to have lower network connectivity in that regional network. Moreover, if a gene has low expression in both regions or high expression in both regions, it does not have a large connectivity difference between the two regional networks. Since we are only analysing genes that have statistically significant differential expression, we can interpret downstream results with more confidence and not expect the results being due to chance. In our analyses on genes with a large difference in their network connectivity, we attribute this to their down regulation in a certain stage of AD because we know that certain regions get affected later on in the course of AD progression and therefore show early molecular changes occurring in AD. However, if genes that are not DE in a region are included in the network, we would not be able to ascertain whether the connectivity difference was due to statistically insignificant changes in expression (either due to technical regions or biological) or because they were truly up or down regulated in that stage of AD.

There has not been much documentation of AD progression in the MTG [63], nor any analysis of the MTG from a systems biology viewpoint. MTG has been thought to play a role in face recognition, which is known to be a latter symptom of AD. Our results suggested that the middle temporal gyrus is not as severely affected as the other three regions. The large physiological differences, evidenced by the significant pathways, and the large difference in gene connectivity between the MTG and the other three regions were the bases for this conclusion. Based on this observation we speculate that post-mortem analyses of the MTG in end-stage AD affected individuals could shed more light onto early AD pathogenesis. Our analyses showed that network topological differences between different brain regions can aid in identifying pathways that differ between regions if there is a large difference in the underlying processes between the regions. Furthermore, the association between a gene's activity and disease severity could be determined by examining its connectivity across the networks.

We did not perform this study expecting the method to show that MTG would be less severely affected. After all the analyses of the different comparisons were completed, we were able to search for processes of interest (such as cytoskeletal, transport, etc.) in the list of DE genes in the SFG and PVC as well as conclude that MTG is not severely affected. Nevertheless, by looking at the DE genes of the 4 AD affected regions one cannot clearly identify which processes to analyse further or reach such a conclusion. Since our conclusions regarding the MTG conform to what is already known from imaging and neuropathological reports, speaks well of the ability of this approach compared to other existing methods. Our results provide additional insights from a genetic and genomic perspective, bridging a gap between genetic information and disease phenotypes, and complement the findings from imaging and neuropathological studies. Furthermore, a conclusion from such analyses is a form of independent and unbiased conclusion. These facts pave the way for applying this novel method to investigate other lesser understood human diseases and conditions, as well as time course analysis, in order to generate testable hypotheses or, the more interesting scenario of comparing mild cognitive impairment (MCI) and AD. This paper has presented the application of this analysis method by applying to a well studied disease with a good microarray dataset so that results can be verified via literature search. Experimental validation of 100 s of genes is neither feasible nor cost effective. Therefore, it would be prudent to first test the method on well studied topics. We are currently applying this method (with good results) to study time-dependent and dose-dependent response to low-dose ionising radiation in a 3-D skin model (manuscript under preparation).

We performed our analyses with the differentially expressed genes between AD affected and normal controls within each region for computational simplicity as well as to have more confidence in the interpretation of results as these DE genes are considered AD-perturbed genes. However, coexpression networks can be built using all the genes on the microarray chip. In order to preserve computational simplicity as well as include as many genes as possible, we used a relaxed FDR of 0.5% to select DE genes so that a larger number of genes could be common to the pairs of regions analysed.

Although we proposed a particular measure of differential network topology, any valid measure of differential topology can be used. The same rationale applies to the TO threshold as well. The rationale is that network differences between data, that are more similar than different, may help in highlighting and understanding the causes of the differences in the data. From this study, it is postulated that methods relating to differential coexpression network topology should be included in the toolkit of techniques employed to study complex diseases such as Alzheimer's.

## Competing interests

The authors declare that they have no competing interests.

## Authors' contributions

MR and WZ conceived and designed the study. MR carried out the computational analysis and performed the biological interpretation. MR and WZ wrote the paper. All the authors read and approved the final manuscript.

## Supplementary Material

Additional file 1 **Number of network links for all regions**. This excel sheet provides the number of network connection for all the genes in the network in each region. Click here for file

Additional file 2 **Six comparisons and twelve coexpression networks of the four regions**. There were six comparisons among four brain regions. The coexpression networks for regions were built using the intersection genes of the regions being compared. The resulting networks built from the intersection genes are shown on the right. Click here for file

Additional file 3 **Lists of TO genes**. This excel sheet lists all the genes with zero topological overlap between regions. Click here for file
